# Supplementary figures and images for: An electrodiffusive neuron-extracellular-glia model for exploring the genesis of slow potentials in the brain
Source: PLoS Comput Biol. 2021 Jul 16;17(7):e1008143. doi: 10.1371/journal.pcbi.1008143 (PMC8318289; doi:10.1371/journal.pcbi.1008143)

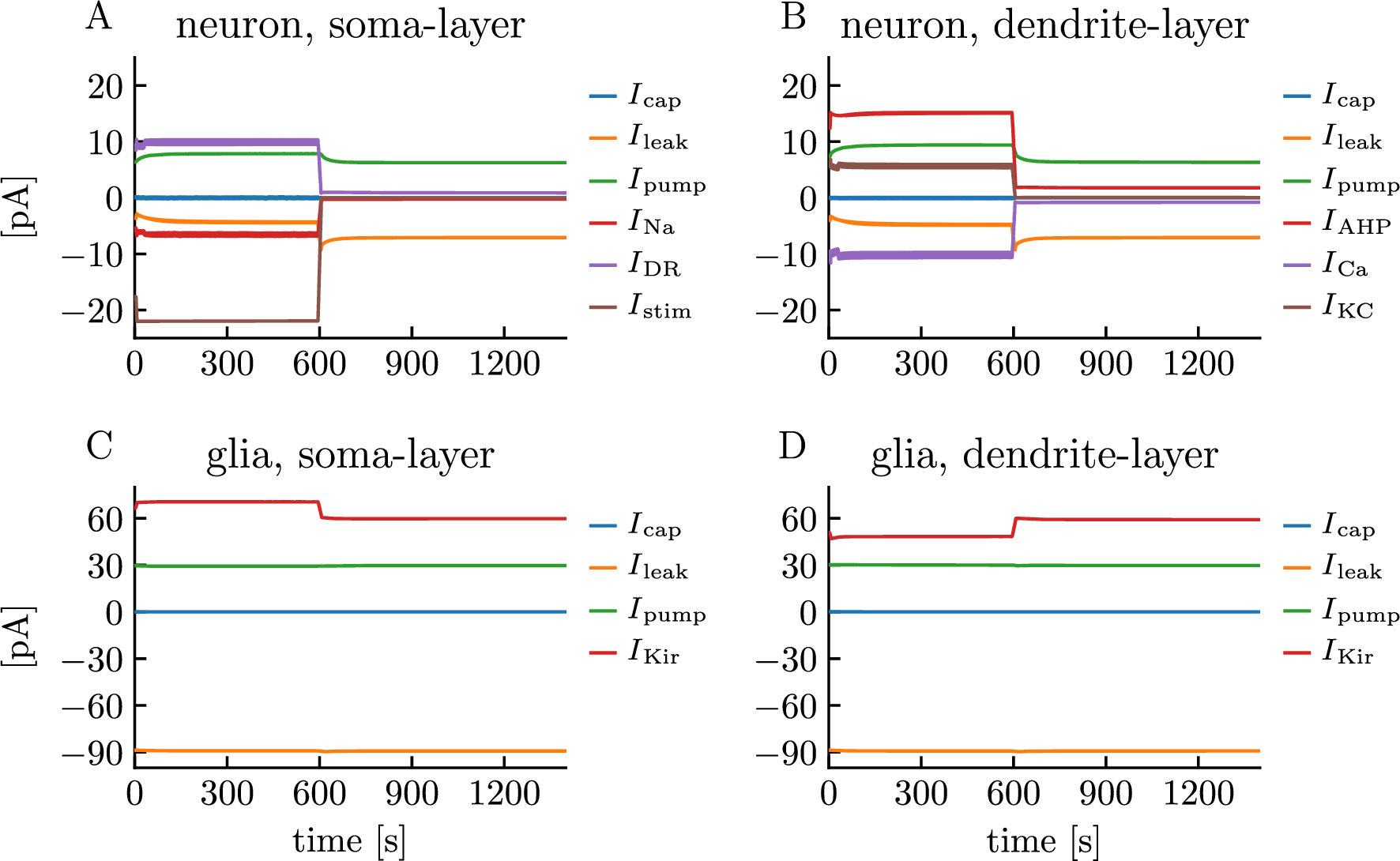

Supplement: S1 Fig — Components (various ion channels, stimulus, capacitive currents, and ion pumps) of the transmembrane current in the neural soma layer (A), the neural dendrite layer (B), the glial soma layer (C), and the glial dendrite layer (D). The current components were plotted as moving averages using a time window of 10 s. The simulation was the same as in Fig 3. (A) The neuronal membrane current was dominated by the injection stimulus current (sink) in the soma-layer during firing. Among the other currents, the delayed rectifying K+ current contributed the most, but the other (ionic) current components were on the same order of magnitude. As expected, the capacitive current (∝ dϕm/dt averaged over 10 s) was close to zero. After firing ceased (t > 600 s), the membrane current was dominated by the pump and leak currents, being oppositely directed, keeping the cell in a steady resting state. (B) The afterhyperpolarizing K+ current dominated the neuronal membrane current (source) in the dendrite-layer during firing, but the other (ionic) currents were on the same order of magnitude. In the steady resting state, the pump- and leak currents dominated the membrane current, just like in the soma-layer. (C-D) In both glial compartments, the (inward) leak current was the largest component, closely, but not entirely, balanced by the (outward) Kir and pump currents. Whether the total membrane current amounted to a glial source (soma-layer) or sink (dendrite-layer) was determined by the magnitude of the Kir current. (TIF) [file pcbi.1008143.s001.tif]

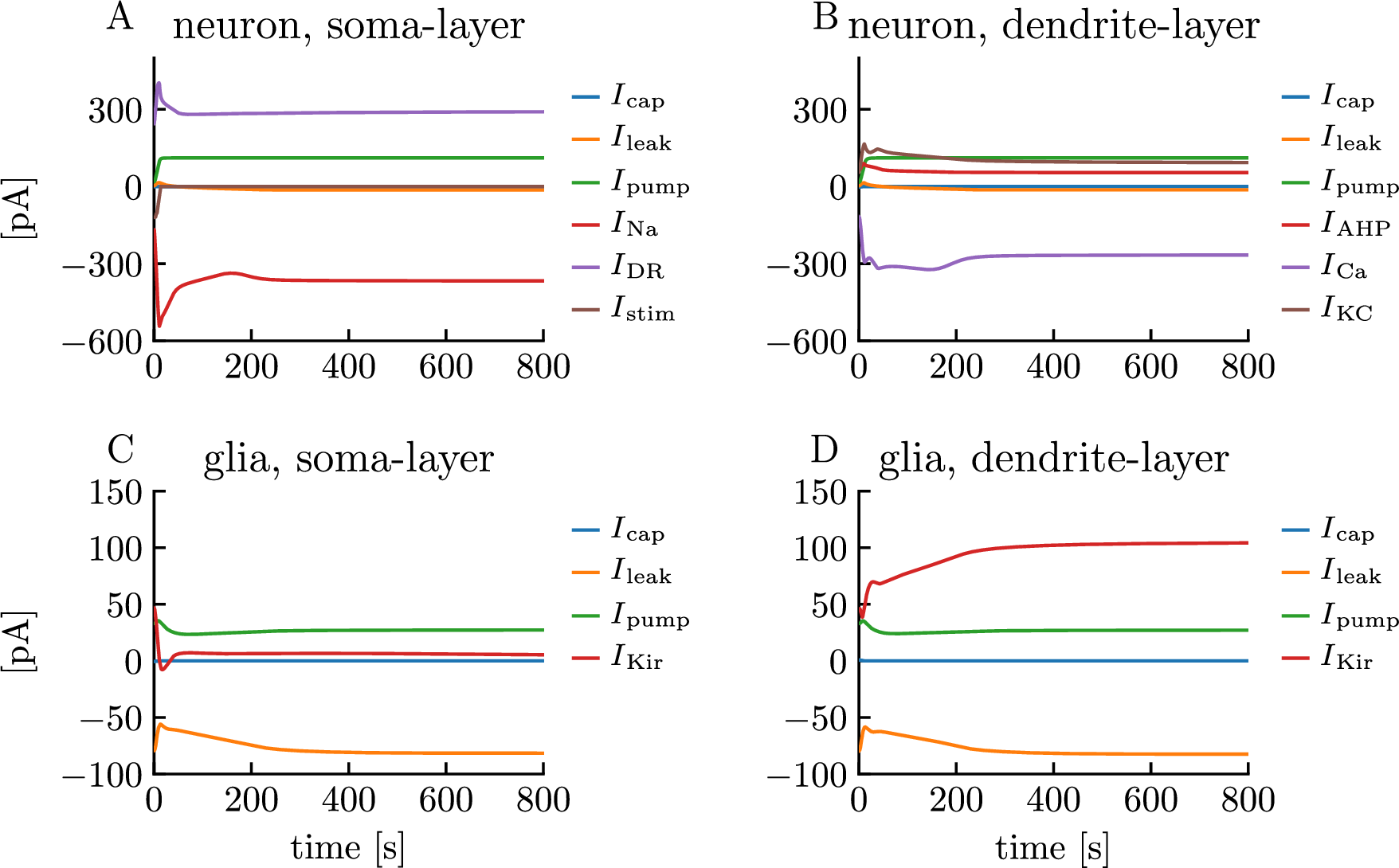

Supplement: S2 Fig — Components (various ion channels, stimulus, capacitive currents, and ion pumps) of the transmembrane current in the neural soma layer (A), the neural dendrite layer (B), the glial soma layer (C), and the glial dendrite layer (D). The current components were plotted as moving averages using a time window of 10 s. The simulation was the same as in Fig 4, where the neuron was driven into depolarization block. (A) At the end (and throughout most) of the simulation, the neuronal membrane current in the soma-layer was primarily composed of an (outward) Na+ current, and the (inward) delayed rectifying K+ current and pump current. The Na+ current was largest in magnitude, but smaller than the sum of the two inward currents, so that the soma was a net current source. (B) At the end of the simulation, the neuronal membrane current (sink) in the dendrite layer was dominated by the (inward) Ca2+ current. (C) The glial membrane currents were dominated by the leak current (sink) in the soma-layer and (D) the Kir current (source) in the dendrite-layer. (TIF) [file pcbi.1008143.s002.tif]
