# Supplementary material for: An electrodiffusive neuron-extracellular-glia model for exploring the genesis of slow potentials in the brain
Source: PLoS Comput Biol. 2021 Jul 16;17(7):e1008143. doi: 10.1371/journal.pcbi.1008143 (PMC8318289; doi:10.1371/journal.pcbi.1008143)
Supplement: Attachment — Submitted filename: Dear Editor.pdf [file pcbi.1008143.s004.pdf]

Dear Editor,

We hereby resubmit our manuscript PCOMPBIOL-D-20-01192 by Marte J. Sætra, Gaute T. Einevoll, and Geir Halnes for publication in PLoS Computational Biology. The manuscript has been revised substantially, and the new version is titled "An electrodiffusive neuron-extracellular-glia model for exploring slow potentials in the brain".

We thank the editor and reviewers for their time and effort. In addition to the reproducibility report (Reviewer 2), we received comprehensive, highly insightful, and very challenging feedback from three reviewers (Reviewers 1,3 and 4). It has taken us quite some time to respond to this feedback, and we indeed think that the manuscript has been substantially improved as a consequence.

All the three reviewers (1,3 and 4) appreciated the modeling framework and its potential value for the research community, but also came with several critical remarks. Taken together, the criticism from the three reviewers pointed in quite a variety of directions, as summarized in the list below:

1. **Single cell models:** It was suggested that we add additional membrane mechanisms, including Ca-channels in the glia cells, I_h, I_A and Na channels on neural dendrites, Ca- and I_AHP currents in the neural soma, and synapses on the neural dendrites. Furthermore, it was suggested that we perform a better characterization of the neural firing properties, and adapt the model to e.g., patch clamp data.

2. **Validation:** It was suggested that we validate the model to data from experiments on spreading depression, with the aim to tune the model to a specific experimental context or to data on how glial effects on neuronal somata might differ from effects on neural dendrites.

3. **Stimulus protocol:** The stimulus protocol (constant K+ influx to the neuron) used to evoke neural firing was criticized, and we were encouraged to use a more realistic (e.g., synaptic) stimulus.

4. **Biophysical modeling:** The model was criticized for neglecting water flow due to hydrostatic water pressures, for using quasi-Ohmic (Hodgkin-Huxley-type) approximations for the membrane currents instead of the Goldman-Hodgkin-Katz equations, and for using the same osmotic model for neural and glial swelling when aquaporin channels are present in glial cells but not neurons.

5. **Geometrical representations:** Questions were raised regarding the geometrical assumptions applied in our model, such as the small number of compartments used to represent the neuron, the volume to surface ratios associated with the neuronal compartments, and the use of closed boundary conditions. It was suggested that we implement open boundaries, or let the system interact with an external bath solution, or let the glial cell transport K+ out of the system in a way accounting for glial siphoning of K+.

6. **Purpose/Hypothesis:** All the reviewers made remarks suggesting that the model was under-exploited, i.e., that we did not use it for a clear purpose, testing a hypothesis regarding for example ephaptic coupling, extracellular potential gradients, or spreading depression under specific experimental contexts.

This is quite a handful, but despite all these critical remarks, the reviewers expressed a general positivity towards the submitted work, giving the model “the very highest recommendation” (R1), referring to it as “reasonable” (R3), and as “a solid piece of work in a well-written and well-documented paper” (R4). The reason why such a positive attitude survived such a long list of critical remarks, is probably the appreciation of the edNEG model as a type of modeling framework that is needed within the field. As it is the first tri-domain type of tissue model that is spatially explicit in the vertical (soma vs dendrite) direction in which the tissue is inhomogeneous, it can be used to examine phenomena involving concentration gradients and slow potential gradients across layers - phenomena that cannot be examined with other existing frameworks.

Inspired by the comments from the reviewers, we have found a clearer application of the model. In the revised manuscript, we have run simulations with the purpose of investigating the generation of slow (DC-like) potentials in brain tissue, and how this depends on mechanisms such as (i) slow currents sources/sinks in neurons, (ii) slow (buffering) current sources/sinks in glial cells, and (iii) extracellular diffusion. This has resulted in a more focused body of work, and we have let the “purpose” determine what is necessary to include in the model.

We were not able to implement all remarks/suggestions (1-6) made by the reviewers. To our defense, we want to emphasize that we consider the main value of the work to be that it proposes a framework that makes it possible to address problems that could not be addressed with previous frameworks, and it puts in place the most important categories of biophysical processes that we believe to be involved in the genesis of slow potentials. We considered the specificities of the system that it was applied to to be of secondary priority in this first implementation, and for simplicity, we therefore chose to re-use a set of neuronal and glial membrane mechanisms from two previously published models. Apart from the fact that we (as reviewers suggested) added synapses to the model, did some re-tuning to obtain a more reasonable Cl- reversal potential and larger extracellular concentration gradients, we stuck with the same cell models as in the original submission. We do not consider this first implementation as an attempt to model a specific biological scenario, but rather as an application to two example cell models that could (possibly) be replaced in future studies in order to compare the effect of different cell types/different ion channels on slow potentials.

Concerning the geometrical representations, it is far from obvious how one best should represent the neuron-extracellular-tissue variations in the vertical direction with a simplified domain-type of geometry. As always when making model abstractions, one must make decisions as to what kind of system properties one wishes to preserve. This is a problem even in standard models, when one for example reduces complex dendritic structures to multicompartment models or ball-and-stick models. Typically, one then collapses the real geometry in a way that preserves electrotonic length constants and certain aspects of somatodendritic signaling, and let the (real) compartment sizes and volume-to-surface ratios be compromised in order to achieve

this. The problem is even more complex in the model that we present here, since it includes both voltage and concentration variables, which scale differently with the volume to surface ratios of cellular compartments. We were not able to deduce any analytical principles as to how this best should be done, but simply specified our geometrical parameters in order to obtain plausible extracellular concentration gradients in the system during normal and pathological neural firing. In the revised version of the manuscript, we have tried to be more clear about these complications, and have expanded the discussion on this topic.

We believe that the revised model includes the main functionality needed for the purpose of exploring the genesis of slow potentials in brain tissue, and hope that the reviewers appreciate our efforts to organize the manuscript so that it better addresses a specific research question.

PLoS Computational Biology encourages authors to mark changes in resubmitted manuscripts by using red text. We have done this in the Methods section, which was only partially changed. However, as the rest of the manuscript was almost entirely rewritten, we have not marked the changes there (it would be all red).

Below follows a detailed response to the points raised by the reviewers, using the color coding:

Blue: Reviewers´ comments.

Black: Authors´ response.

Red: Citations of added/edited text in the revised version of the manuscript.

Reviewer #1:

R1.1: The manuscript "An electrodiffusive neuron-extracellular-glia model with somatodendritic interactions” by Satra, Gaute T. Einevoll and Geir Halnes uses numerical simulations of electrodiffusion model to investigate the spatial-temporal communication between neuron and glia cells.

Following the logic and results of the manuscript, I guess that the most important achievement is a model itself. Readers can find a new electrodiffusion model of glia - neuron intercommunication via intra- and extracellular ionic concentrations. The suggested model is realistically representing the complex phenomenon in a space between neuron and glia and, from my point of view, can be the essential step in the definition of existing hidden conversations between cells living in the electrolyte.

The main benefit of the manuscript is the model itself: attractive, well-described, with details mechanisms based on principles of electrodiffusion, including critical parameters of geometrical configurations. The authors highlighted critical limitations of the model also.

Theorists will benefit the manuscript if accepted, will actively cite and use as desk information when someone needs to find the right equation or the correct parameter.

The work is accompanied by program code in Python, which adds advantage for this article. Everyone simulating with Neuron will be happy to include some of the scripts to their models.

My opinion is that the model deserves the very highest recommendation. The model is in some way similar to the already published by authors “An electrodiffusive, ion conserving Pinsky-Rinzel model with homeostatic mechanisms Marte J Sætra, Gaute T Einevoll, Geir Halnes”.

A1.1. We very much appreciate this positive evaluation of our work.

R1.2. The drawback of this project, like many other similar projects, is the triviality of the results. The authors first wrote a new exciting model, and then began to think about what to do with it, and not vice versa. For example, after discovering a new phenomenon that cannot be explained using the old paradigm authors have proposed a new theory (model) that explains the new phenomena.

However, for the sake of fairness, in general, the results may be of interest to a wide range of readers working in the field of electrolyte dynamics, cell biophysics, and electrophysiology.

To improve the manuscript, the authors should explicitly specify a working scientific hypothesis and identify the main scientific results of the project. The paper should have a logical organisation that reflects the aims or research questions of the manuscript, including any hypotheses that have been tested. The model results needs to make connections with experimental data.

A1.2. As we argued in the introductory part of this letter, the edNEG model represents a new type of framework - one that can be used to examine phenomena involving slow potential gradients in the vertical (soma to dendrite) direction in tissue that cannot be examined using other existing frameworks. We believe that such a framework has value in itself, as it can lend itself to many possible applications.

That being said, we agree that the edNEG model (and many other models) would benefit from being presented in combination with an application to a well defined research question. We have taken your remarks into account, and in the revised version we use the edNEG model to study the genesis of slow potentials in brain tissue. We think that the manuscript has become a much more exciting read as a consequence, so we thank you for your suggestion.

We admit that the model is currently on a fairly abstract/qualitative stage. That is, we have chosen to make this initial implementation of it using two (rather simple) previously published cell models, but not tuned it in order to represent a particular biological system or brain region. A direct connection with data can therefore not be made, but we do compare the model predictions (when made) with typical values reported in the literature.

R1.3. A few comments on the text and the artwork

Introduction.

R1.3.1. Lines 38-39. The statement “Until recently, models that were consistent in this regard (see, e.g., [16, 27{29]), had not 39 accounted for morphological aspects of neurons “ is not correct, please see the paper of J. Cartailler, D. Holcman Electrodiffusion Theory to Map the Voltage Distribution in Dendritic Spines at a Nanometer Scale Neuron, 2019 Nov 6;104(3):440-441. doi: 10.1016/j.neuron.2019.10.025.

A1.3.1. You are correct. There exist several spatially explicit models that are consistent in this regard, and we did not state clearly that we were speaking of tissue-level domain-type models. We have now rephrased the sentence (lines 80-83) to:

Domain-type models that are consistent in this regard (see, e.g., [37–39]), have not accounted for the differential expression of membrane mechanisms in dendrites versus somata, or do not include a glial domain [33].

We have also included a passage in the Discussion (lines 516-521) where we make the distinction between domain-type and spatially explicit models. The paper that you suggested was included among the cited works:

Several electrodiffusive models have been developed for systems with explicit geometries (see e.g., [32,101–107]. These models have the advantages that they are conceptually easy to interpret, i.e., the various compartments (often finite elements) exist side by side in a spatially explicit meaning. However, they have the disadvantage that they are very computationally demanding, and applications have so far been limited to processes taking place on rather small spatial and temporal scales.

R1.3.2. Fig.1 Authors defines volume fractions as 0.4/0.2/0.4. Since this is a critical parameter of the electrodiffusion model that will able to change results, would be nice to have an explain of this numerical value and the citations here are critical.

A1.3.2. We agree and have now included a reference to Chen and Nicholson (2000) in Table 1, Geometrical parameters.

R1.3.3. Fig.2. To compare two models A-C and B-D please plott results at the same time frames?

A1.3.3. This figure is no longer part of the paper.

R1.3.4. Fig.3. “The glial domain did not contain any Ca2+ channels.” Glia has a lot of Ca channels, why authors excluded the Ca channels from glia?

A1.3.4. It is true that glia cells have Ca2+ channels, and Ca2+ is important for a number of intracellular processes going on in glial cells. However, we expect that transmembrane Ca2+ currents are comparably small compared to K+, Na+, and Cl- currents. Furthermore, Ca2+ is the least abundant ion among those included in the model. We therefore do not believe that Ca2+ channels in glia cells would have any major impact on the slow potentials that we focused on in the study.

R1.3.5. Table 1. I recommend micrometres for dimension. Why geometrical parameters in the table, which in general have no physical measurement, are

deduced with such precision, and not just 1000, 500 and so on. Was there any meaning in that or the value for some model calculations, stability, for example.

A1.3.5. The rationale behind using SI base units for dimensions was to maintain consistency between equations, tables, and code. We chose to stick with this, although we are aware that it is more common within the field to list geometrical parameters with units of micrometres and square centimetres.

The table values stem from our previous study, Sætra et al. 2020, where we used intracellular volumes and membrane areas corresponding to spheres with radius 7 μm, and a Δx that is half of the region thickness. We have added the following text to the table caption to clarify this:

We assumed a region thickness of 1.3 μm and consequently a Δx that was half of this. The cellular compartment volumes and membrane areas correspond to spheres with radius 7 μm.

R1.3.6. Eq. 118 and 119, is it a typo?

A1.3.6. Thanks for pointing this out. This is now fixed and the equations are numbered 131 and 132. We have also replaced the section named "Stimulus current" with the sections "Injection current" and "Synaptic current".

R1.3.7. The question related to the equations 4-9. I was not able so to found how the authors solve the problem associated with electrodiffusion in conditions of non-uniform geometry, for example, in case of the transition from the soma to the dendrite, the place where the number of ions can be preserved, but not concentrations since a jump of geometry. In this case, membrane potential will also be jumped, which leads to a non-uniform electric lateral current.

A1.3.7. The membrane potential is a function of the total charge in a compartment divided by the membrane area, and is as such determined by the number of ions (not the concentration). We can assure you that the model was implemented in such a way that it ensured that bulk electroneutrality was preserved under all circumstances (we ran several tests on this). We hope that this answered your question.

R1.3.8. The Eq.63 and 64. The electrical current of any co-transport, Itr, is determined by product of a membrane conductivity and a deviation of membrane potential from the Nernst potential, Itr=G(V-Enernst). However, in the manuscript, The current of co-transporters (Eq 63) is the multiplication of conductivity by the Nernst potential and thus does not depend on the membrane potential, and Eq 64 depends on membrane potential but in a nonlinear way. Please explain.

A1.3.8. We apologize, but are not sure that we understand your question. Eq. 64 (in the original submission) for the Na/K/2Cl cotransporter did not depend on the membrane potential. While currents through ion channels are driven by the Nernst-potential, pumps and co-transporters operate through other kinds of (more complex) mechanisms, with dependencies on other variables than the ion channels. In any case, all the membrane mechanisms were taken from previous models, and none of these were invented in the current work.

R1.3.9. I would recommend rewriting equations 70-73 with the right-hand side explicitly dependenting on a volume.

A1.3.9. This was a good idea, and we did as suggested. These equations are now numbered 116-121.

Reviewer #2: Reproducibility report has been uploaded as an attachment.

A2.1. Following the reviewer(s) suggestion, we have applied an open source licence (MIT) to the published code.

Reviewer #3:

R3.1. The paper proposes a framework for modeling of neuronal excitation and ionic dynamics in a macroscopically homogeneous neural tissue that includes neurons, glial cells and extracellular space (ECS). Because of importance of spatial extension of neurons, the neurons are considered as two-compartmental ones surrounded by different subpopulations of glial cells and different compartments of ECS. Such 2-by-3 compartment description seems to be well equilibrated. The authors introduce a convenient set of notations for main variables and formulate the model with canonical equations of electrical circuits and ionic channels, as well as with a phenomenological approximation of volume change. Such formulation pretends to be a standardized one, and seems to be useful for programming.

A3.1. Thanks for this positive feedback.

Concerns:

R3.2. I agree that the distinction of somatic and dendritic compartments is critically important. The reasons (not mentioned in the manuscript) are: (i) comparison and reproduction of experimental patch-clamp recordings in voltage- and current-clamp modes require at least 2-comp. model; (ii) calculation of local field potentials requires at least 2-comp. model; (iii) ionic dynamics is different in somatic and dendritic compartments of neurons, because of inhomogeneous distribution of synaptic receptors. I also agree that the glial cells adjacent to different neuronal compartments may show different dynamics and are probably worth to be distinguished. Thus, the proposed 2x3-model seems to be reasonable. However, synaptic interaction between neurons is much more powerful in sense of contribution to both the neuronal excitation and the ionic dynamics. Unfortunately, the synapses are absolutely ignored in the paper, which devaluates the work.

A3.2. We agree, and have added synapses to the revised version of the model. The synaptic currents are defined under the Synaptic current section in Methods, and simulations based on synaptic stimuli are shown in the new Figure 7.

R3.3. In its present form, the model seems to be just a slightly updated version of the very recently published model of the same authors [17]. The key moment of their model description is an integral form of the equations for voltages. Including glia is straightforward and known, in particular, from their previous work [38]. Its effect has been shown before. A novelty of the present paper is two compartments of glia, but the authors do not refer to those effects of soma- and dendrite-adjacent glial compartments that could be revealed in experiments.

A3.3. It is true that the edNEG model builds heavily on the two previous models [17] & [38]. However, we want to point out that it is not entirely trivial to combine them. Since the neuron and glia cell will interact through a shared extracellular space, the new system will find a new equilibrium, and in fact, when we combined the two (without making any other changes), the two cells ended up with completely unrealistic resting potentials. Hence, (as we explain in the Methods sections under Model tuning) we needed to find a new set of initial ion concentrations and leak conductances to make sure that the full system had a reasonable equilibrium.

We do, however, agree that many of the simulation results obtained on the updated model overlapped with those presented in the previous work [17]. In the revised version, we have redefined the objectives of the work, and focused it on the (previously un-simulated) interplay between neurons, glial cells, and diffusion in the context of generating slow extracellular potentials.

R3.4. The model does not take into account the lateral diffusion of ions and their diffusion in the bath solution. The latter is quite necessary for comparison with slice experiments, however, it may destroy the conservative form of the equations.

A3.4. This is true. We chose to use closed boundary conditions, because it has a clear interpretation that is meaningful in the context of studying slow extracellular potentials. As we argue in the results section (line 332-340):

--- the closed boundary conditions applied in the simulations are equivalent to assuming periodic boundary conditions in the horizontal direction. Fig 5 thus simulates the extracellular spike in the hypothetical case of a population of perfectly synchronized neurons, i.e., one where all neurons in some brain region are identical and fire an AP at the exact same time. While such synchrony is unreasonable on the short time scale of AP firing, it seems like a reasonable assumption for processes on a longer time scale (arguments for this were also given in [15]). Synchrony in slow signal components of, say, 0.1 Hz, simply means that all the neurons have the same average activity over a 10 s period.

The topic of how to choose boundary conditions was revisited in the Discussion (lines 570-575), where we add to the above, and also include your suggestion that a setup with open boundaries would be better suited to model slice experiments:

An alternative, and equally simple setup would be to use open boundaries, for example allowing the extracellular domain to be coupled with a constant bath-solution. Such a setup was not tested in the current implementation of the edNEG model, but could perhaps be valuable if one wished to mimic conditions with localized input to a neuron

in an otherwise silenced region, and could putatively be used to model various slice experiments.

R3.5. The initial results are focused on Ca-spikes, whereas the later results are on the model of spreading depression (SD) in the absence of synaptic activity, i.e., presumably, the case of experiments with zero-Ca solution. I see a contradiction between these two targets.

A3.5. As mentioned previously in this letter, we did not aim to match this first implementation of the modeling framework to any specific experimental setup. However, two reviewers suggested that we implement synaptic activity and this is now studied in Fig 7.

R3.6. Stimulation of the neuron with only potassium current (Eqs.118 and 119), as if with a pure potassium pump, seems to be unrealistic.

A3.6. The motivation for using an injection current of pure K+ was that a previous computational study showed that stimuli with K+ caused less physiological disruption (Kneller et al. 2002) than stimulus using other ion species. We have now added this reference on lines 773-775 in the Methods section:

If nothing else is stated, we applied a K+ injection current to the soma. A previous computational study of a cardiac cell showed that K+ ions cause least physiological disruption when used as stimulus [67].

However, we agree that this is not fully realistic, and have now studied the neuron under different stimulus regimes, including synaptic input (see Figs 6 and 7).

R3.7. The authors consider as an advantage of the model that it reproduces spike shapes obtained in rather old Pinsky&Rinzel's modeling work. To my opinion, those shapes are not common, if searching within recent patch-clamp recordings.

A3.7. In the revised manuscript, we have kept our focus on investigating slow extracellular potentials, and we have therefore removed the parts addressing the spike shapes in the neural model. We do not believe the spike shape to be of any major importance for the slow extracellular potentials, which depend on membrane currents averaged over larger time windows. Probably, the Pinsky-Rinzel model is as plausible as any model for the use in this context, and we chose to keep it as it is.

R3.8. Instead, it would be valuable to validate the simulated effects of ionic concentration shifts on neuronal activity with recent experiments.

A3.8. The effects of ionic concentration shifts on neural activity has been the topic of many computational studies, typically using single compartment neuron models, interacting with a single extracellular compartment. We cited a selection of these works in the introduction (lines 55-57):

Electric ephaptic effects have been the topic of many studies (see, e.g., [21–28]), as has the effect of changing ion concentrations on neurodynamics (see, e.g., [29–33]).

In the revised version of the manuscript, we have removed most of the simulations that focus on short term neural activity, and focused on the slow potentials. We are not aware of any experimental data that could be used easily for direct validation of our results, although our re-defined project was motivated by experimental work reporting a correlation between neural hyperactivity, increased extracellular K+ concentrations, and slow potentials. In lines 14-17 of the introduction we cite a small selection of these works:

Slow potentials are often correlated with changes in extracellular ion concentrations, and especially with rises in the extracellular K+ concentration. Such correlations are, for example, found regularly in studies of seizure activity [4,5] and spreading depression [6,7].

R3.9. Am I right that the low firing frequency (about 1Hz) of the neuron is due to the AHP-channel (beta_q=1)? If so, it seems to be unrealistic. A plot of spiking frequency versus current and shunting conductance (or current at least) would be helpful to characterize the neuron.

A3.9. You are right that the AHP-channel affects the neuronal firing rate, but we want to point out that the low firing frequency seen in Fig 3 also is due to the weak stimulus current (22 pA). If being stimulated with a strong injection current, the neuron is able to fire in steady-state with a firing frequency of up to 25 Hz. It can also tolerate higher firing frequencies than this for shorter periods of time. Since our focus in this paper was the modeling framework and its potential to describe the genesis of slow potentials, we chose to not study the single cell characteristics.

R3.10 The model considers ``a local piece of neuro-glial brain tissue", and any considerable shift of ionic concentrations in ECS is expected to originate not from a single neuron but from many neurons in some vicinity. However, as written, for instance, in the ``Homeostatic breakdown in the edNEG model" section, only a single neuron is stimulated: ``There, the neuron received a strong input current…". I guess, the authors mean the stimulation of all neurons simultaneously, which relates to Figs.3-6.

A3.10. We agree that this was unclear in the originally submitted manuscript. As we state clearly in the revised version, we do consider this to be a tri-domain model, describing the average activity (all cells) in a unit-piece of tissue. In the revised version we explain early in the results section that the reference to neurons and glial cell should be understood within a tri-domain context:

To keep notation short, we will in the following refer to the neural, ECS, and glial domains, simply as the neuron, the ECS, and the glia cell.

To clarify how the model should be interpreted, we also state (Results section, line 332-340) that the way in which the model is set up is equivalent to having periodic boundary conditions, i.e., conditions where all neurons within a local piece of tissue have identical dynamics:

--- the closed boundary conditions applied in the simulations are equivalent to assuming periodic boundary conditions in the horizontal direction. Fig 5 thus simulates

the extracellular spike in the hypothetical case of a population of perfectly synchronized neurons, i.e., one where all neurons in some brain region are identical and fire an AP at the exact same time. While such synchrony is unreasonable on the short time scale of AP firing, it seems like a reasonable assumption for processes on a longer time scale (arguments for this were also given in [15]). Synchrony in slow signal components of, say, 0.1 Hz, simply means that all the neurons have the same average activity over a 10 s period.

R3.11. It is written concerning the depolarization block that ``This kind of dynamics can not be captured with standard neuron models constructed under the assumption that ion concentrations remain constant under the simulated period." It is not true.

A3.11. We agree that it was imprecise. We should have specified that we talked about the dynamical interplay where shifts in ion concentrations drives the neuron into depolarization block. This sentence didn't survive the revision.

R3.12. The authors do not observe recovery from depolarization block. They suggest that ``the recovery might be observed if the edNEG model were expanded to a spatially continuous model". I guess, it is sufficient to take into account the potassium relaxation to its concentration in the bath solution, as done in many previous models, like in [44] and [68].

A3.12. We agree also on this and have now added this to the sentence. It now says (lines 256-260):

As the edNEG model studied here represented a local and closed system, such spatial riddance of K+ did not occur, but we anticipate that recovery might be observed if the edNEG model was expanded to a spatially continuous model or if K+ was allowed to "leak" out of the system, relaxing towards its concentration in an external bath solution

R.3.13. Synaptic currents provide important contributions to SD, at least NMDAR-mediated currents (Somjen 2001). These currents provide one of the major sources of sodium and potassium ionic fluxes. Therefore, it is not correct to simulate the ionic dynamics during SD without taking NMDA-currents into account. (Though, it has been done in some previous works.) In its present version the model might pretend to reproduce some slice recordings under the conditions of synaptic receptor blockage and equal for all neurons excitation, presumably, provided with optogenetics.

A3.13. In the revised version of the manuscript, we no longer claim to simulate spreading depression, although we still believe that the model, or at least some adapted version of it, could be useful within such a context.

That being said, synaptic input might also play an important role for the slow potentials that we have focused on in the revised manuscript, and is, of course, more physiologically realistic than constant current injections. We have therefore followed your suggestion and simulated input conditions with NMDA-synapses (Fig. 7) in addition to the ones using constant current injections (Fig. 6).

P.S. see our replies above (**A3.4.** and **A3.10.**) for system interpretations, and possible adaptations of the model to simulate slice experiments.

R3.14. Panels A and C of Fig.6 demonstrate the effect of glia. However, if the potassium concentration in glia changes only because of K-IR channels and the pump, then in a stationary regime the total potassium flux into glia is zero, and thus the glia should not affect the potassium concentration in ECS. Neglecting by the volume change, I conclude that the glia just occupies some volume thus reducing the ECS volume. So, the effect of glia seems to be trivial, it is just an effect of a single parameter, which is the ratio of neurons' to ECS's volumes.

A3.14. In the edNEG model, volumes and areas are defined explicitly, with the initial ECS volume being half as big as the neuronal volume. This is the case whether the glia cell is present or not. We agree that the K+ exchange must be zero in the steady-state, just like the K+ exchange across the neuronal membrane must be zero in the steady-state. However, neither the neuron nor the glia cell is in steady-state when they start to fire or when the neuron goes into the depolarization block. Furthermore, we show in the revised manuscript that the glia cell contributes to the extracellular potential, meaning that a net current must leave one of the compartments and enter the other compartment. For example, the slow extracellular potentials during steady state firing or depolarization block are due to slow steady-state current loops through both the glial cell and the neuron (see, e.g., Figure 6).

R3.15. The authors explain AHP observed in Fig.6B by the effect of the pump and compare with the experiment from Gulledge et al. 2013 [49]. Though the magnitude of the effect seems to be similar (a few mV), it is not the case. The AHP in [49] takes place in the resting, low-conductance state, whereas similar voltage shift in Fig.6B is observed in the regime of high conductance. This fact points to overestimation of the pump density in the model.

A3.15. This figure is no longer part of the paper, and since we have not observed this AHP in any other figure, neither in the old or revised manuscript, we have not followed up on this.

R3.16. Spreading depression is a spreading phenomenon, but it is modeled as a localized event, which, however, is a common place of models.

A3.16. See our reply above (**A3.13**).

R.3.17. The volume dynamics is driven by the osmotic pressure. At the same time, it is known that neurons do not have aquaporins (Murphy // Front. Cell. Neurosc. 2017) and react to osmotic pressure change weaker than to the change of potassium concentration [Andrew et al. // Cer.Cortex 2007]. The choice of the osmose-based model for the volume dynamics in neurons is not justified.

A3.17. You are right that neurons do not have aquaporins, but water can still cross the membrane through the lipid bilayer, and most likely through non-aquaporin channels [Andrew et al. // Cer.Cortex 2007, Boss et al. // Journal of Biomedical Optics 2013]. It has been shown that neurons respond to osmotic gradients [Boss et al. // Journal of Biomedical Optics 2013] and that they swell dramatically upon prolonged depolarization [Andrew et al. // Cer.Cortex 2007]. The assumption of osmose-based transport of water across the neuronal membrane is applied in several previous models, see eg. [Mori 2015, Tuttle et al. 2019, Kager 2002]. However, the absence of

aquaporins makes neurons less permeable to water compared to astrocytes, which is reflected in our parameter choices.

R3.18. The model neglects the flux of water between somatic and dendritic compartments of both neurons and ECS. Why? The reference to [95], where the macroscopic flow is considered, seems to be irrelevant here.

A3.18. We realized from your comment that our reference to Holter et al. was not sufficient to justify our modeling choice. In fact, intra-domain water flow might play an important role in brain function. However, there is still controversy about its magnitude, underlying mechanisms, and even its presence [Mori // Physica D 2015]. We have now removed the old reference to Holter et al., an added the following paragraph:

We only considered effects of transmembrane water flow, and intra-domain water flow due to hydrostatic pressures were neglected.The assumption is applied in several previous multi-compartment models, e.g. [35, 37, 80], but as Mori et al. [35] point out, intra-domain water flow in brain tissue is not fully understood and may play an important role in brain function. It is an ongoing area of computational research, see e.g. [103, 104], and the edNEG model may contribute to this work if the framework gets extended.

R3.19. The constructed model is aimed to provide ``a general framework for combining multicompartmental neural modeling with electrodiffusive ion concentration dynamics in neuroglial brain tissue", whereas ``many previous models have parts of the same functionality". Below I have listed some most representative works of different teams of authors and compared the details included and not included in their models.

[79] – Krishnan et al. 2015 – net, 2 comp. + glia + KCC2 + propagation in 1D + synapses, but not syn. contribution in the ionic dynamics, no transporters

[68] Wei et al. 2014 "Oxygen…" – network, + synapses +volume, but not syn. contribution in the ionic dynamics

[44] Wei et al. 2014 "Unification…" – 1 comp. + glia + KCC2, NKCC1 + volume + O2

Gentiletti and Suffczynski // IJNS 2017 – E and I, 3 comp. + glia, but no synapses

Chizhov et al. // Plos One 2019 – network, 2 comp. + glia + NKCC1, KCC2 + synapses contributing to ion dynamics, but no spatial propagation, no volume

[13] Kager et al. 2000 – distributed neuron + buffer + K-dependent NMDA

[60] Florence et al. 2009 - 1 comp. + glia, but no synapses, no transporters, no spatial propagation

[75] Chang et al. 2013 – 2-comp. + glia + O2 + blood + propagation in 1D, but no synapses, no transporters

[27] Tuttle et al. 2019 – 1-comp. + glia + propagation in 2D + NMDA contributing to ion dynamics + volume, but no network

Unfortunately, in its present version the constructed model also has only parts of the target functionality.

A3.19. You are right. We did not express ourselves clearly there. We have rephrased the description of what are the unique properties of the proposed framework, which now reads (lines 473-477):

However, to our knowledge, the edNEG model is the first tri-domain type of electrodiffusive tissue model that is spatially explicit in the vertical (soma → dendrite) direction, and therefore the first framework that can be used to simulate phenomena involving concentration gradients and slow potential gradients across layers in a self-consistent manner.

Two of the works that you cited above were new to us, and we have cited them along with the other list of models with "parts of the same functionality".

Minor comments:

R3.20. Abbreviation KNP appears in Results before its introduction in Methods.

A3.20. Thank you for spotting this mistake. It has been corrected.

R3.21. Black square in Fig. 3A looks weird.

A3.21. To make it less weird, we have added an extra panel (B) showing the shape on an action potential (zoomed in-view of panel A).

R3.22. One of the labels ``I" in the caption to Fig.6 should be ``J".

A3.22. You are right. However, this figure is no longer part of the paper.

R3.23. Formulas relating a concentration to an amount of substance and a volume is given only in words (after eq.10), which is inconvenient for readers.

A3.23. We have added the appropriate equations (see Eqs 10-15).

R3.24. "Constraint number (iv)" should be `` Constraint number (4)" before eq.17.

A3.24. We have corrected this (see line 631).

R3.25. Hodgkin-Huxley approximations are written as dependent everywhere on the voltage phi_m. Should it be phi_{dn} for the dendritic currents and phi_{sn}-phi_{se} for the somatic currents?

A3.25. You are absolutely right. Something similar goes for the Ca2+ concentration in Eqs 49, 65, and 66, and we have revised the manuscript to correct this. To keep notation short, we used phi_msn for the somatic membrane potential and phi_mdn for

the dendritic membrane potential. These are defined on lines 619 and below Eqs 43 and added to the Model summary (Eqs 112-115).

R3.26. Similar, the volume in eq.66; the voltage and concentrations in eqs.67-69.

A3.26. Since Eqs 68-72 (neuronal stabilizing mechanisms) apply to both neuronal compartments, we have kept the old notation so that we don't have to write the equations twice, but we now clarify in the text below that *n* denotes the neuronal compartment and *e* denotes the extracellular compartment. Same applies to Eqs 73-75 (glial stabilizing mechanisms) where we now clarify that *g* denotes the glial compartment and *e* denotes the extracellular compartment.

R3.27. M is not given in eq.76.

A3.27. M is now explicitly defined and inserted into Eqs 78-81 (Volume dynamics).

Reviewer #4:

R4.1. The authors present a computational model of a (layered) two-compartment neuron (soma-dendrite) and (two) glia cells connected both to a two compartmental extracellular space. The neuron and the glia cell have a standard collection of ionic conductances and ionic pumps/exchangers that allow not only to calculate the dynamics of electrical currents and membrane voltage, but also all ionic concentrations and fluxes between the compartments. They demonstrate in their results that this model is "stable" at a defined resting state, the neuron can fire and goes into depolarization block already at a relatively low firing rate of ~8 Hz. They also incorporated the consequences of changing osmotic pressure as a consequence of ion changes in the compartments: swelling and shrinking of those compartments under "normal" physiological conditions and under the extreme pathological condition of spreading depression.

The authors present a solid piece of work in a well-written and well-documented paper. I think the model they present is not as new as they claim (almost any aspect of it has already been presented in published work by others); however this model has the potential to add substantially to our knowledge of the functional consequences of neuron-glia interactions and the role of ion(concentration)s in the nervous system. One of the properties in which this model can surpass the already published (NEURON environment) models is that it is capable of calculating the extracellular potential(gradient)s. Unfortunately, the authors only mention this in a hidden sentence and do not expand it into something useful (see below, under compartments). The main body of the paper describes the architecture of the model, composed of the combination of two well-validated models of a neuron and a glia cell. The validation of the complete model in the results is not very strong and more an illustration than a test against known experimental facts. More than half of the discussion is spent on presenting potential new applications of the model in analyzing spreading depression. If this part of the paper would be moved to the results and seriously designed and investigated, it would considerably enhance the impact of this paper/model.

A4.1. Thank you for your positive evaluation of our work. We are also grateful for the constructive criticism, which inspired us to make the study of (slow) extracellular potentials the main focus of the revised manuscript. We believe that this has improved the manuscript significantly.

R4.2. The proposal in the discussion to use the tri-domain-model as a basic element to create a larger system (and also investigate the spreading in "spreading depression") is suggestive, but also a huge oversimplification of what needs to be done. The model, however, is potentially better capable of investigating ephaptic interactions between neurons than many of the existing ones. For the currently presented single neuron situation the extracellular potential gradients are too small to be relevant, however we know that in regions like CA1, CA3 and DG in the hippocampus we encounter extracellular field potentials of several millivolts, well capable of affecting the phenomena under study here.

A similar remark holds for the glia cell. The way the coupling is now proposed in the tri-times two domain model in the discussion does not pay tribute to the power of spatial glia buffering. The current model is implemented as a "closed system", but we know that for the phenomenon under study (spreading depression) K+ is virtually transported to the EC outside the realm of the neuron by the glia syncytium: very strongly electrically coupled groups of glia cells. In a more primitive implementation K+ can be temporarily buffered in the blood stream; a mechanism that is certainly relevant for the spreading of SD and again a realm where this model could fill an interesting spot.

My strong suggestion is to at least incorporate one of these aspects in the result section.

A4.2 You are correct: To embed the tri-domain model as a basic element within a larger system would probably be a quite demanding modeling project. We do believe that such a modeling project would indeed be meaningful, and do have some ideas regarding what needs to be done to accomplish it. However, your remarks convinced us to remove this proposal from the current manuscript and to let the model stand more on its own.

Inspired by your comments, we have now obtained larger concentration gradients in the extracellular space and do indeed see ephaptic interactions. As we explain in the Methods section (lines 736-741), the previous version of the model had a very fast redistribution of ions in the extracellular space, so that almost no concentration gradients developed between the soma and dendrite. In the revised version, we obtained larger concentration gradients and diffusion potentials by reducing the cross-section area for extracellular fluxes (and currents). The new Figure 5 shows an ephaptic coupling between the neuron and glial cell.

A4.3. I also suggest that the authors are more careful in calling stabilizing mechanism homeostatic: I prefer to reserve the latter word for mechanisms that at least seem to have some kind of "set-point" that they try to maintain. Most mechanism that are mentioned in this paper under homeostatic have indeed a stabilizing effect, but to the best of my knowledge they do not try to preserve a defined set-point. In fact the authors need to tweak the "leak currents" in order to

attain a stable resting membrane voltage. This is often done in this type of modelling and I do not object. However critical questions about the physiological meaning of these ion-selective non voltage-dependent channels cannot be answered. Calling them leak is to a large extend hiding the problem!

A4.3. We have now changed the wording from homeostatic to stabilizing when talking about individual mechanisms (and not the homeostatic machinery as a whole).

R4.4. The adequacy of a model and its correctness can never be judged without taking into consideration its detailed purpose. As far as I can judge this model is supposed to present a realistic firing pattern for a CA3/CA1 hippocampal neuron and should contain the elements necessary to describe the depression aspect of the phenomenon of "spreading depression". The authors suggest that the model can be universally applied to solve many questions. However, for that aim at least a few elements need to be added (and probably a motivation why they are not incorporate would clear things up and warn potential users!).

A4.4. Although we still believe that spreading depression is one phenomenon that the edNEG models could shed light upon, we have shifted the focus away from this pathological phenomenon. Also, as we indicated in the initial part of this letter, we did not put an emphasis on modelling the spiking repertoire of any particular neuron in any detail, but on constructing a framework for exploring extracellular slow potentials produced by any desired combination of cells. In this first implementation of this framework, we chose to adopt two previously published cell models, and used them in their original form.

R4.4.1. The transient A-current and the hyperpolarization activated h-current are two potassium currents with a very important functional role in the firing patterns of these pyramidal neurons.

A4.4.1 We do not want to make the claim that our neuron model is highly representative for a particular cell type. The main importance is the qualitative aspect that it has differential expression of ion channels in the soma versus dendrites. We comment further on the possible inclusion of A-currents and h-currents when replying to one of your other remarks (see **A4.5.5** below)

R4.4.2. It is unclear why there is no Na current in the dendrites: this prevents any realistic study on phenomena where back-propagating APs are relevant.

A4.4.2. In line with our replies (**A4.4** and **A4.4.1**) above, this modeling choice was adopted from the original model by Pinsky & Rinzel. In the original Pinsky-Rinzel model (as in our adopted and ion conserving version), the coupling between the dendritic and somatic compartments was sufficiently strong for somatic APs to give quite large voltage deflections in the dendrites. Adding (active) Na channels in the dendrites would boost this effect, but would probably only give rather minor quantitative changes in our predictions of slow potentials. However, as we have argued earlier, we saw a value in re-using the original model in its original form for this study, since our focus is not on the details regarding neuronal spiking properties.

R4.4.3. The lack of any Ca current in the soma (maybe even the low and the high threshold variants) and an IC-AHP K current in the soma might be the reason that we see hardly any of the normally observed frequency adaptation in these neurons. In my opinion these currents need to be present if one wants to undertake a study of depolarization block. All pyramidal cells that I have recorded from easily produced firing rates in the 10-20 Hz range without ever going into depolarization block.

A4.4.3. We think that the low firing tolerance seen in the previous version of the manuscript (Fig 6) was an effect of the stimulus protocol (strong step-pulses, each evoking one AP), and not an intrinsic property of the model itself. When given a continuous step current, the neuron can fire with a firing frequency of 25 Hz, or even higher if it is only for a short period of time. We add an example simulation over 30 seconds:

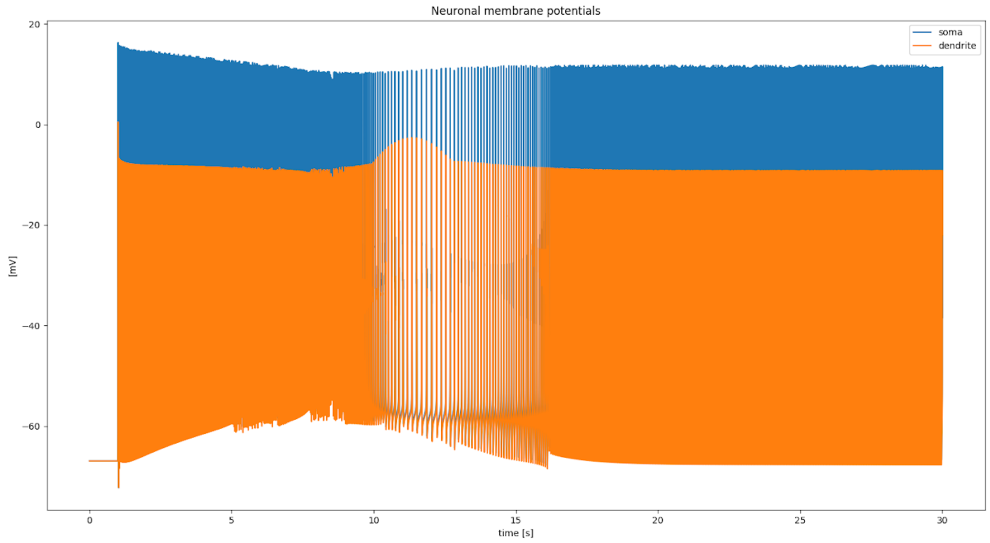

R4.4.4. The implementation of the model implies a reversal potential for chloride in rest of -90.3 mV. That is extremely low and not supported by experimental evidence. As there are no chloride channels it also assumes a very high chloride pumping rate in the rest situation.

A4.4.4 We agree that the chloride reversal potential in the previously submitted model version was unrealistically low. As we explain in lines 709-716 of the methods section, we have now re-tuned and re-calibrated the model so that the chloride reversal potential is -79 mV in the neuron.

R4.4.5. It would be helpful for the experimentalists if the authors reported the input-impedance of their neuron and the glia cell in the resting state.

A4.4.5 We measured the input resistances of the cells and found an input resistance of 540 MOhm for the neuron and 48 MOhm for the glia cell. Since we do not put too much focus on the neuronal (or glial) response properties, we chose to not report these values in the manuscript.

R4.5. Whether or not the above-mentioned omissions/additions are relevant, strongly depend on the phenomena one wants to describe and the precision one wants to attain. More serious are the points mentioned below as they deal with fundamental errors in the model:

A4.5. In the revised version, we have used the edNEG model to explore the genesis of slow extracellular potentials, and have based our modeling choices on what is relevant for this endeavor.

R4.5.1. As is done very often in modeling the authors use the Nernst equation to calculate the reversal potential and then Ohm's law to describe the ionic current. This is only correct in the equilibrium situation. Currents here should be calculated using the Goldman-Hodgkin-Katz equation. I do not think that this will strongly affect the conclusions, but it will considerably change the numbers. If that is not relevant, why then do we try to make correct models?

A4.5.1 Our main objective when constructing the edNEG model was to define a framework that ensures a consistent relationship between all currents, all concentrations and all voltages in all compartments. The framework should be compatible with any set of membrane mechanisms, provided that currents were ion specific. Our best argument for staying with the quasi-Ohmic, Hodgkin-Huxley (HH) type membrane currents, was that we chose to use the Pinsky-Rinzel model, and therefore wanted to keep the membrane mechanisms in the same form as there. You are right that the use of GHK would be more accurate from a biophysical point of view, and that it might change neural spike shapes, especially for Ca2+ spikes. Such, putatively small, alterations in membrane currents would probably alter the firing properties of the Pinsky-Rinzel model, at least to some degree, and would force us to conduct a new tuning project to restore its original firing properties. We did not want to put that much emphasis on the tuning of firing properties model tuning in this work, as this would take the focus away from our main topic. As argued earlier, we saw the value in keeping the membrane currents on their previously published form.

R4.5.2. The authors use an (artificial) K current injection into the soma to depolarize the neuron and bring it to firing. In real life firing is mostly brought about by synaptic activation, implying a substantial change in synaptic conductance with current flow and depolarization as a consequence. This looks like a minor difference, but the change in conductance affects the membrane time constant and thus the dynamics of the currents. It also implies that most activity starts from the dendritic compartment (although during SD, the shifts in ionic composition will directly initiate somatic firing). In the current model synaptic conductances are not implemented, but there is hardly a phenomenon where they are not involved….

A4.5.2 Synapses are now added to the model (Fig 7). See **A3.6.** above.

R4.5.3. The authors assume the same volume fractions for the somatic and the dendritic compartments, which is clearly not the case and experimentally well documented. It might explain why they notice little difference in their soma versus dendritic observations (fig 5).

A4.5.3. As we discussed in the introductory part to this response letter, representing the neuron-extracellular-tissue variations in the vertical direction (soma vs dendrite) with a simplified domain-type geometry is far from trivial, and the interpretation of what volume to surface ratios mean in this context is not obvious. The use of identical somatic and dendritic compartments was in the edNEG model a heritage from the Pinsky-Rinzel model, but you are correct that this is a model parameter that could be modified with the aim to reproduce specific data from a specific brain region. We chose not to do so here, but have added a fairly long discussion on the topic of selecting geometrical parameters (see lines 515-547 in Discussion.)

Also, in the revised model, we have changed cross section area for extracellular currents and particle fluxes, and by doing that, we now do have more pronounced differences between somatic and dendritic observations (Fig 3 and 4 in the new manuscript).

R4.5.4. A similar remark holds for the substantial difference that should exist between the somatic surface-volume ratio, compared to the dendritic surface-volume ratio. There are a few vague remarks on this point, but the consequence should be a considerable difference in current-inflow to concentration change in the two regions (probably hidden because the lack of Ca^{2+} influx in the soma and synaptic ionic influx in the dendrites).

A4.5.4. This, and your previous remark, are two issues that we have thought a lot about. It is an interpretational problem that we still struggle with, and you do make a good point.

In an early stage of this modeling project, we did try to select geometrical parameters that preserved the experimentally reported surface to volume ratios in brain tissue. The problem with doing that was that the dendritic trees had enormous surface to volume ratios. A single compartment, for example a sphere, preserving that surface to volume ratio would be like a tiny evaporating black hole. When collapsing the neural domain into two compartments, we could not preserve this ratio and at the same time obtain a reasonable coupling between the somatic and dendritic activity. The reason is partly that the dendritic "sensing" of the somatic activity in reality decays with distance, an aspect that is not accounted for when collapsing the whole tree into a single compartment. As we discussed in the introductory part of this letter, the challenge in representing neurons with an abstract geometry is related to (but much more complex than) the model-reduction problems extensively studied by Rall, i.e., how to collapse complex dendritic structure into simplified models including just a few compartments. Following Rall's recipe, the real surface-to-volume ratios are compromised in order to preserve the voltage-dynamics properties of the neurons, reflected through the electrotonic length constant calculated for the dendrites. In our case, we do not only have voltages, but also ion concentrations to worry about, and without having any analytical derivations to refer to, we have realized that the electrotonic length constant is not at all the same as the "chemical" length constant - i.e. electric signals spread differently through dendrites than ion fluxes, and so on. Hence, we do not have any analytical guidance as to how such a model reduction should be done in a systematic manner. For simplicity, we therefore assumed that the dendritic and somatic compartments had identical geometries (this was also the case in the original Pinsky-Rinzel model, although compartment volume had no significance there). Apart from

this, we set geometrical parameters rather freely to values that gave us fairly reasonable output on the neurodynamics and on the extracellular concentration (and slow potential) gradients. We thought that this was the best that we could do, given our current state of knowledge.

This being said, a more systematic exploration of how changes in e.g., the dendritic surface area (or volume) could change the model predictions would be a good topic for a follow-up study.

R4.5.5. The authors claim that two compartments in the neuron catch the essentials of the neuron's behavior, but also that adding more compartments is not a big deal. The latter improvement is needed if we want a better inside into the extracellular potential gradients and if we want to implement IA and Ih which have a specific gradient distribution in the dendrites of these cells. Such expansions are relatively easy done in modeling environments as NEURON, but a lot harder in Python code!

A4.5.5. The processes studied with the edNEG model could not be studied using NEURON or other standard simulation software. However, it is correct that model expansions are much simpler in these softwares than in our Python code, so we have rephrased this part, and discussed the possible inclusion of dendritic ion channels with gradient distributions. Lines 506-514 of the Discussion now read:

Many ion channels, such as non-specific hyperpolarization activated cation channels (Ih) and A-type K+ channels (IA) often have gradient distributions in neural dendrites [98], and to explore effects of this it might be necessary to expand the edNEG model by including additional compartments for each of the domains. Such an expansion would require a re-writing of the model code, but would not require any fundamental changes of the conceptual framework that it was based upon, and should therefore not be too challenging for a user with training in programming.

R4.5.6. The authors attain conservation of particles in their model but have a huge problem to attain electroneutrality in all compartments. They solve it by adding impermeable anions which is OK. However they need 97% of the anions in the neuron to be impermeant and 95% in the glia, which is far more than experiments suggest. They also add 16 mM impermeant anions to the extracellular space, for which there is no experimental support whatsoever, to the best of my knowledge…

A4.5.6. We do not believe this to be a main concern with the model, but merely a technicality used to obtain electroneutrality. It is well established that the intracellular solution in cells is indeed electroneutral (or at least extremely close to electroneutral). The intracellular concentrations of the ions that we keep track of in our model (Na+, K+, Cl- and Ca2+) were reasonable, i.e., in the same ballpark as standard values (see e.g., table 2.1 in Somjen 2001 (10 mM Na+, 125 mM K+, 6.6 mM Cl-, and a very small Ca2+ concentration). Since those values do not add up to a zero charge, there must by necessity be additional negative charge present. In reality, this charge is there in the form of macromolecules (possibly with high negative valencies) or other ions not included in the simulations, such as for example, HCO3-, for which Somjen 2001 reports a concentration of 18 mM. In the model, we ensured electroneutrality by adding a number of anions with valency -1, and additional uncharged molecules to balance osmotic pressure gradients at rest. These ions do not affect the simulations, and we

might have achieved the same equilibrium through other choices (e.g., using half the number of divalent anions). These static charges are static in the sense that we do not model their dynamics, but we do not mean to make any claims about whether or not they in reality are static or do not permeate the membrane.

R4.5.7. I understand that the purpose of the illustrations in the results is more to illustrate than to validate against experimental data, but is about 0.5 mM increase in extracellular K+ for each AP of a single neuron not a bit much? (Fig 3B).

A4.5.7. In the previous version, we did not plot ion concentrations, but ionic reversal potentials. In the revised version, we have plotted the extracellular ion concentrations and as seen in Fig 3, the extracellular K+ concentration increases by about 0.1 mM for each AP. That might still seem a bit much. However, as we explained earlier (see our reply **A3.10.** above), this does not represent the effect of a single AP in a single neuron, but rather the effect that we would see if all neurons in a piece of tissue fired an AP in perfect synchrony. 0.1 mM might be a reasonable value for such a (hypothetical) scenario. We have included a passage in the revised manuscript (lines 330-340) where we explain this issue.
