# Supplementary material for: An electrodiffusive neuron-extracellular-glia model for exploring the genesis of slow potentials in the brain
Source: PLoS Comput Biol. 2021 Jul 16;17(7):e1008143. doi: 10.1371/journal.pcbi.1008143 (PMC8318289; doi:10.1371/journal.pcbi.1008143)
Supplement: Attachment — Submitted filename: PlosCB_revision_SaetraEinvollHalnes.pdf [file pcbi.1008143.s005.pdf]

Comments to the revised paper

``An electrodiffusive neuron-extracellular-glia model with somatodendritic interactions”

by Marte J. Saetra, Gaute T. Einevoll and Geir Halnes

Please, find below my commentaries written in green.

Reviewer #3:

R3.1. The paper proposes a framework for modeling of neuronal excitation and ionic dynamics in a macroscopically homogeneous neural tissue that includes neurons, glial cells and extracellular space (ECS). Because of importance of spatial extension of neurons, the neurons are considered as two-compartmental ones surrounded by different subpopulations of glial cells and different compartments of ECS. Such 2-by-3 compartment description seems to be well equilibrated. The authors introduce a convenient set of notations for main variables and formulate the model with canonical equations of electrical circuits and ionic channels, as well as with a phenomenological approximation of volume change. Such formulation pretends to be a standardized one, and seems to be useful for programming.
A3.1. Thanks for this positive feedback.

Concerns:

R3.2. I agree that the distinction of somatic and dendritic compartments is critically important. The reasons (not mentioned in the manuscript) are: (i) comparison and reproduction of experimental patch-clamp recordings in voltage- and current-clamp modes require at least 2-comp. model; (ii) calculation of local field potentials requires at least 2-comp. model; (iii) ionic dynamics is different in somatic and dendritic compartments of neurons, because of inhomogeneous distribution of synaptic receptors. I also agree that the glial cells adjacent to different neuronal compartments may show different dynamics and are probably worth to be distinguished. Thus, the proposed 2x3-model seems to be reasonable. However, synaptic interaction between neurons is much more powerful in sense of contribution to both the neuronal excitation and the ionic dynamics. Unfortunately, the synapses are absolutely ignored in the paper, which devaluates the work.
A3.2. We agree, and have added synapses to the revised version of the model. The synaptic currents are defined under the Synaptic current section in Methods, and simulations based on synaptic stimuli are shown in the new Figure 7.
RR3.2. The simulations in Fig 7 do not present any synaptic interactions and neuronal excitation affected by ionic dynamics and determined by the synaptic interactions.

R3.3. In its present form, the model seems to be just a slightly updated version of the very recently published model of the same authors [17]. The key moment of their model description is an integral form of the equations for voltages. Including glia is straightforward and known, in particular, from their previous work [38]. Its effect has been shown before. A novelty of the present paper is two compartments of glia, but the authors do not refer to those effects of soma- and dendrite-adjacent glial compartments that could be revealed in experiments.
A3.3. It is true that the edNEG model builds heavily on the two previous models [17] &[38]. However, we want to point out that it is not entirely trivial to combine them. Since the neuron and glia cell will interact through a shared extracellular space, the new system will find a new equilibrium, and in fact, when we combined the two (without making any other changes), the two cells ended up with completely unrealistic resting potentials. Hence, (as we explain in the Methods sections under Model tuning) we needed to find a new set of initial ion concentrations and leak conductances to make sure that the full system had a reasonable equilibrium. We do, however, agree that many of the simulation results obtained on the updated model overlapped with those presented in the previous work [17]. In the revised version, we have redefined the objectives of the work, and focused it on the (previously un-simulated) interplay between neurons, glial cells, and diffusion in the context of generating slow extracellular potentials.

RR3.3. This is my main concern. I did not describe it well in my previous review. The equation for the membrane voltage in the form of the capacitor charge relation C*U=Q is inapplicable. Indirectly it follows from the commentary R4.5.6 written by Reviewer 4. This commentary was not properly satisfied. Number of ions needed to charge the membrane-capacitor up to the voltage about 70mV is 5 orders of magnitude less that the total number of ions in the neuron. So, any realistic, i.e., approximate values of ionic concentrations cannot determine the voltage. As a consequence, the authors need this artificial number of impermeable anions, 97%.

R3.4. The model does not take into account the lateral diffusion of ions and their diffusion in the bath solution. The latter is quite necessary for comparison with slice experiments, however, it may destroy the conservative form of the equations.
A3.4. This is true. We chose to use closed boundary conditions, because it has a clear interpretation that is meaningful in the context of studying slow extracellular potentials. As we argue in the results section (line 332-340):
--- the closed boundary conditions applied in the simulations are equivalent to assuming periodic boundary conditions in the horizontal direction. Fig 5 thus simulates the extracellular spike in the hypothetical case of a population of perfectly synchronized neurons, i.e., one where all neurons in some brain region are identical and fire an AP at the exact same time. While such synchrony is unreasonable on the short time scale of AP firing, it seems like a reasonable assumption for processes on a longer time scale (arguments for this were also given in [15]). Synchrony in slow signal components of, say, 0.1 Hz, simply means that all the neurons have the same average activity over a 10 s period.
The topic of how to choose boundary conditions was revisited in the Discussion (lines 570-575), where we add to the above, and also include your suggestion that a setup with open boundaries would be better suited to model slice experiments:
An alternative, and equally simple setup would be to use open boundaries, for example allowing the extracellular domain to be coupled with a constant bath-solution. Such a setup was not tested in the current implementation of the edNEG model, but could perhaps be valuable if one wished to mimic conditions with localized input to a neuron in an otherwise silenced region, and could putatively be used to model various slice experiments.
RR3.4. The limitation of the model has been described in the text. The model has not been changed.

R3.5. The initial results are focused on Ca-spikes, whereas the later results are on the model of spreading depression (SD) in the absence of synaptic activity, i.e., presumably, the case of experiments with zero-Ca solution. I see a contradiction between these two targets.
A3.5. As mentioned previously in this letter, we did not aim to match this first implementation of the modeling framework to any specific experimental setup. However, two reviewers suggested that we implement synaptic activity and this is now studied in Fig 7.
RR3.5. Ok

R3.6. Stimulation of the neuron with only potassium current (Eqs.118 and 119), as if with a pure potassium pump, seems to be unrealistic.
A3.6. The motivation for using an injection current of pure K+ was that a previous computational study showed that stimuli with K+ caused less physiological disruption (Kneller et al. 2002) than stimulus using other ion species. We have now added this reference on lines 773-775 in the Methods section:
If nothing else is stated, we applied a K+ injection current to the soma. A previous computational study of a cardiac cell showed that K+ ions cause least physiological disruption when used as stimulus [67].
However, we agree that this is not fully realistic, and have now studied the neuron under different stimulus regimes, including synaptic input (see Figs 6 and 7).
RR3.6. Still, the simulations can hardly remind any experiments.

R3.7. The authors consider as an advantage of the model that it reproduces spike shapes obtained in rather old Pinsky&Rinzel's modeling work. To my opinion, those shapes are not common, if searching within recent patch-clamp recordings.
A3.7. In the revised manuscript, we have kept our focus on investigating slow extracellular potentials, and we have therefore removed the parts addressing the spike shapes in the neural model. We do not believe the spike shape to be of any major importance for the slow extracellular potentials, which depend on membrane currents averaged over larger time windows. Probably, the Pinsky-Rinzel model is as plausible as any model for the use in this context, and we chose to keep it as it is.

RR3.7. Ok

R3.8. Instead, it would be valuable to validate the simulated effects of ionic concentration shifts on neuronal activity with recent experiments.
A3.8. The effects of ionic concentration shifts on neural activity has been the topic of many computational studies, typically using single compartment neuron models, interacting with a single extracellular compartment. We cited a selection of these works in the introduction (lines 55-57):
Electric ephaptic effects have been the topic of many studies (see, e.g., [21–28]), as has the effect of changing ion concentrations on neurodynamics (see, e.g., [29–33]).
In the revised version of the manuscript, we have removed most of the simulations that focus on short term neural activity, and focused on the slow potentials. We are not aware of any experimental data that could be used easily for direct validation of our results, although our re-defined project was motivated by experimental work reporting a correlation between neural hyperactivity, increased extracellular K+ concentrations, and slow potentials. In lines 14-17 of the introduction we cite a small selection of these works:
Slow potentials are often correlated with changes in extracellular ion concentrations, and especially with rises in the extracellular K+ concentration. Such correlations are, for example, found regularly in studies of seizure activity [4,5] and spreading depression [6,7].
RR3.8. So experiments are mentioned only in the Introduction. The problem of the work is that it is not targeted to any certain experiments.

R3.9. Am I right that the low firing frequency (about 1Hz) of the neuron is due to the AHP-channel (beta_q=1)? If so, it seems to be unrealistic. A plot of spiking frequency versus current and shunting conductance (or current at least) would be helpful to characterize the neuron.
A3.9. You are right that the AHP-channel affects the neuronal firing rate, but we want to point out that the low firing frequency seen in Fig 3 also is due to the weak stimulus current (22 pA). If being stimulated with a strong injection current, the neuron is able to fire in steady-state with a firing frequency of up to 25 Hz. It can also tolerate higher firing frequencies than this for shorter periods of time. Since our focus in this paper was the modeling framework and its potential to describe the genesis of slow potentials, we chose to not study the single cell characteristics.
RR3.9. Ok

R3.10 The model considers ``a local piece of neuro-glial brain tissue", and any considerable shift of ionic concentrations in ECS is expected to originate not from a single neuron but from many neurons in some vicinity. However, as written, for instance, in the ``Homeostatic breakdown in the edNEG model" section, only a single neuron is stimulated: ``There, the neuron received a strong input current…". I guess, the authors mean the stimulation of all neurons simultaneously, which relates to Figs.3-6.
A3.10. We agree that this was unclear in the originally submitted manuscript. As we state clearly in the revised version, we do consider this to be a tri-domain model, describing the average activity (all cells) in a unit-piece of tissue. In the revised version we explain early in the results section that the reference to neurons and glial cell should be understood within a tri-domain context:
To keep notation short, we will in the following refer to the neural, ECS, and glial domains, simply as the neuron, the ECS, and the glia cell.
To clarify how the model should be interpreted, we also state (Results section, line 332-340) that the way in which the model is set up is equivalent to having periodic boundary conditions, i.e., conditions where all neurons within a local piece of tissue have identical dynamics:
--- the closed boundary conditions applied in the simulations are equivalent to assuming periodic boundary conditions in the horizontal direction. Fig 5 thus simulates the extracellular spike in the hypothetical case of a population of perfectly synchronized neurons, i.e., one where all neurons in some brain region are identical and fire an AP at the exact same time. While such synchrony is unreasonable on the short time scale of AP firing, it seems like a reasonable assumption for processes on a longer time scale (arguments for this were also given in [15]). Synchrony in slow signal components of, say, 0.1 Hz, simply means that all the neurons have the same average activity over a 10 s period.
RR3.10. So, the model describes "the average activity (all cells) in a unit-piece of tissue". Are neurons distinguished by types, like excitatory and inhibitory, at least? If even not, how one can use the proposed set of equations for modeling synaptically interacting populations of neurons? This is the main goal, isn't it? With the present equations one can simulate only spikes from a single neuron that

represents a population of neurons. However, there is a big gap between the spikes and a firing rate that governs the synaptic activity.

R3.11. It is written concerning the depolarization block that ``This kind of dynamics cannot be captured with standard neuron models constructed under the assumption that ion concentrations remain constant under the simulated period." It is not true.
A3.11. We agree that it was imprecise. We should have specified that we talked about the dynamical interplay where shifts in ion concentrations drives the neuron into depolarization block. This sentence didn't survive the revision.
RR3.11. Ok

R3.12. The authors do not observe recovery from depolarization block. They suggest that ``the recovery might be observed if the edNEG model were expanded to a spatially continuous model". I guess, it is sufficient to take into account the potassium relaxation to its concentration in the bath solution, as done in many previous models, like in [44] and [68].
A3.12. We agree also on this and have now added this to the sentence. It now says (lines 256-260):
As the edNEG model studied here represented a local and closed system, such spatial riddance of K+ did not occur, but we anticipate that recovery might be observed if the edNEG model was expanded to a spatially continuous model or if K+ was allowed to "leak" out of the system, relaxing towards its concentration in an external bath solution
RR3.12. The limitation of the model has been described in the text. The model has not been changed.

R.3.13. Synaptic currents provide important contributions to SD, at least NMDAR mediated currents (Somjen 2001). These currents provide one of the major sources of sodium and potassium ionic fluxes. Therefore, it is not correct to simulate the ionic dynamics during SD without taking NMDA-currents into account. (Though, it has been done in some previous works.) In its present version the model might pretend to reproduce some slice recordings under the conditions of synaptic receptor blockage and equal for all neurons excitation, presumably, provided with optogenetics.
A3.13. In the revised version of the manuscript, we no longer claim to simulate spreading depression, although we still believe that the model, or at least some adapted version of it, could be useful within such a context. That being said, synaptic input might also play an important role for the slow potentials that we have focused on in the revised manuscript, and is, of course, more physiologically realistic than constant current injections. We have therefore followed your suggestion and simulated input conditions with NMDA-synapses (Fig. 7) in addition to the ones using constant current injections (Fig. 6).
P.S. see our replies above (**A3.4.** and **A3.10.**) for system interpretations, and possible adaptations of the model to simulate slice experiments.
RR3.13. Ok

R3.14. Panels A and C of Fig.6 demonstrate the effect of glia. However, if the potassium concentration in glia changes only because of K-IR channels and the pump, then in a stationary regime the total potassium flux into glia is zero, and thus the glia should not affect the potassium concentration in ECS. Neglecting by the volume change, I conclude that the glia just occupies some volume thus reducing the ECS volume. So, the effect of glia seems to be trivial, it is just an effect of a single parameter, which is the ratio of neurons' to ECS's volumes.
A3.14. In the edNEG model, volumes and areas are defined explicitly, with the initial ECS volume being half as big as the neuronal volume. This is the case whether the glia cell is present or not. We agree that the K+ exchange must be zero in the steadystate, just like the K+ exchange across the neuronal membrane must be zero in the steady-state. However, neither the neuron nor the glia cell is in steady-state when they start to fire or when the neuron goes into the depolarization block. Furthermore, we show in the revised manuscript that the glia cell contributes to the extracellular potential, meaning that a net current must leave one of the compartments and enter the other compartment. For example, the slow extracellular potentials during steady state firing or depolarization block are due to slow steady-state current loops through both the glial cell and the neuron (see, e.g., Figure 6).
RR3.14. Ok

R3.15. The authors explain AHP observed in Fig.6B by the effect of the pump and compare with the experiment from Gulledge et al. 2013 [49]. Though the magnitude of the effect seems to be similar (a few mV), it is not the case. The AHP in [49] takes place in the resting, low-conductance state,

whereas similar voltage shift in Fig.6B is observed in the regime of high conductance. This fact points to overestimation of the pump density in the model.
A3.15. This figure is no longer part of the paper, and since we have not observed this AHP in any other figure, neither in the old or revised manuscript, we have not followed up on this.
RR3.15. Ok

R3.16. Spreading depression is a spreading phenomenon, but it is modeled as a localized event, which, however, is a common place of models.
A3.16. See our reply above (**A3.13**).
RR3.16. Ok

R.3.17. The volume dynamics is driven by the osmotic pressure. At the same time, it is known that neurons do not have aquaporins (Murphy // Front. Cell. Neurosc. 2017) and react to osmotic pressure change weaker than to the change of potassium concentration [Andrew et al. // Cer.Cortex 2007]. The choice of the osmose-based model for the volume dynamics in neurons is not justified.
A3.17. You are right that neurons do not have aquaporins, but water can still cross the membrane through the lipid bilayer, and most likely through non-aquaporin channels [Andrew et al. // Cer.Cortex 2007, Boss et al. // Journal of Biomedical Optics 2013]. It has been shown that neurons respond to osmotic gradients [Boss et al. // Journal of Biomedical Optics 2013] and that they swell dramatically upon prolonged depolarization [Andrew et al. // Cer.Cortex 2007]. The assumption of osmose-based transport of water across the neuronal membrane is applied in several previous models, see eg. [Mori 2015, Tuttle et al. 2019, Kager 2002]. However, the absence of aquaporins makes neurons less permeable to water compared to astrocytes, which is reflected in our parameter choices.
RR3.17. Ok

R3.18. The model neglects the flux of water between somatic and dendritic compartments of both neurons and ECS. Why? The reference to [95], where the macroscopic flow is considered, seems to be irrelevant here.
A3.18. We realized from your comment that our reference to Holter et al. was not sufficient to justify our modeling choice. In fact, intra-domain water flow might play an important role in brain function. However, there is still controversy about its magnitude, underlying mechanisms, and even its presence [Mori // Physica D 2015]. We have now removed the old reference to Holter et al., an added the following paragraph:
We only considered effects of transmembrane water flow, and intra-domain water flow due to hydrostatic pressures were neglected.The assumption is applied in several previous multi-compartment models, e.g. [35, 37, 80], but as Mori et al. [35] point out, intra-domain water flow in brain tissue is not fully understood and may play an important role in brain function. It is an ongoing area of computational research, see e.g. [103, 104], and the edNEG model may contribute to this work if the framework gets extended.
RR3.18. Ok

R3.19. The constructed model is aimed to provide ``a general framework for combining multicompartmental neural modeling with electrodiffusive ion concentration dynamics in neuroglial brain tissue", whereas ``many previous models have parts of the same functionality". Below I have listed some most representative works of different teams of authors and compared the details included and not included in their models.
[79] – Krishnan et al. 2015 – net, 2 comp. + glia + KCC2 + propagation in 1D + synapses, but not syn. contribution in the ionic dynamics, no transporters
[68] Wei et al. 2014 "Oxygen…" – network, + synapses +volume, but not syn. contribution in the ionic dynamics
[44] Wei et al. 2014 "Unification…" – 1 comp. + glia + KCC2, NKCC1 + volume + O2 Gentiletti and Suffczynski // IJNS 2017 – E and I, 3 comp. + glia, but no synapses
Chizhov et al. // Plos One 2019 – network, 2 comp. + glia + NKCC1, KCC2 + synapses contributing to ion dynamics, but no spatial propagation, no volume
[13] Kager et al. 2000 – distributed neuron + buffer + K-dependent NMDA
[60] Florence et al. 2009 - 1 comp. + glia, but no synapses, no transporters, no spatial propagation
[75] Chang et al. 2013 – 2-comp. + glia + O2 + blood + propagation in 1D, but no synapses, no transporters
[27] Tuttle et al. 2019 – 1-comp. + glia + propagation in 2D + NMDA contributing to ion dynamics + volume, but no network

Unfortunately, in its present version the constructed model also has only parts of the target functionality.

A3.19. You are right. We did not express ourselves clearly there. We have rephrased the description of what are the unique properties of the proposed framework, which now reads (lines 473-477):
However, to our knowledge, the edNEG model is the first tri-domain type of electrodiffusive tissue model that is spatially explicit in the vertical (soma → dendrite) direction, and therefore the first framework that can be used to simulate phenomena involving concentration gradients and slow potential gradients across layers in a selfconsistent manner.
Two of the works that you cited above were new to us, and we have cited them along with the other list of models with “parts of the same functionality”.

RR3.19. Still, it seems that the constructed model has only parts of functionality that is necessary to simulate phenomena involving concentration gradients and synaptic interactions of different type neurons (see my complains **RR3.2 and RR3.10)**, so it is not quite self-consistent.
