# Supplementary material for: An electrodiffusive neuron-extracellular-glia model for exploring the genesis of slow potentials in the brain
Source: PLoS Comput Biol. 2021 Jul 16;17(7):e1008143. doi: 10.1371/journal.pcbi.1008143 (PMC8318289; doi:10.1371/journal.pcbi.1008143)
Supplement: Attachment — Submitted filename: Dear Editor.pdf [file pcbi.1008143.s007.pdf]

Dear editor and reviewers,

We hereby resubmit our manuscript PCOMPBIOL-D-20-01192R1 "An electrodiffusive neuron-extracellular-glia model for exploring slow potentials in the brain” by Marte J. Sætra, Gaute T. Einevoll, and Geir Halnes for publication in PLoS Computational Biology.

We thank the four reviewers for evaluating our manuscript a second time. Reviewers 1, 2, and 4 were satisfied with our new (and quite substantially revised) version of the manuscript. Reviewer 4 suggested some additional improvements, and to a large degree we have followed these suggestions. Reviewer 3 expressed a more critical attitude towards the modeling effort in general, and pointed out several limitations with the model, however, without explicitly demanding that any changes were made to it. We have not changed the model to accommodate these remarks, mainly because we would not know how to do it. Judging from some of the remarks, it appears to us as if reviewer 3 is asking too much from the model. Admittedly, the model is a crudely simplified abstraction of a local piece of brain tissue, and there are many limitations to the kind of problems that can be addressed using it. The question is then whether the model still can be valuable, i.e., if it can provide insights into phenomena that cannot be addressed with other models, and if it provides a tool that can be used in future modeling studies. We believe that it is.

Replies to specific comments are given below, using the color coding:

Blue: Reviewers’ comments.
Black: Our comments.
Red: Citations of new text in the revised manuscript.

Reviewer #1:

The manuscript "An electrodiffusive neuron-extracellular-glia model with somatodendritic interactions” by Satra, Gaute T. Einevoll and Geir Halnes has been improved, and now it is easier to follow the logic and ideas of the manuscript. I think that the result is of interest for experts in the area that deals with simulation of astrocyte-neuron interactions.

I have no technical objections to the publication of the results.

I gratitude the authors for access to the programming code via an online repository Github, to better understand the model.

We thank you for this positive evaluation of our manuscript.

Reviewer #2:

The Reproducibility Report has been uploaded as an attachment.

We thank the reviewer for this useful report. Based on the other reviewers’ comments, we have made changes to the code following Figure 7 and added code for the supplementary material S1 and S2.

Reviewer #3:

The review is uploaded as an attachment

The review was uploaded as a separate attachment, and consisted of direct comments to our answers given in the previous reply letter. Below, we have pasted in the comments that needed an answer, and have tried to provide some additional context (i.e., references to the previous review comments/answers) when necessary. For the full context, see our previous reply letter.

RR3.2. The simulations in Fig 7 do not present any synaptic interactions and neuronal excitation affected by ionic dynamics and determined by the synaptic interactions.

AA3.2. This is true. We consider the response of the neuron (and resulting ion concentration dynamics) to synaptic activation, but do not model synaptic communication between neurons in a network. We would not know how to use a model of this (mass-average) kind to simulate network effects in any meaningful way, and this was not our objective.

RR3.3. This is my main concern. I did not describe it well in my previous review. The equation for the membrane voltage in the form of the capacitor charge relation C*U=Q is inapplicable. Indirectly it follows from the commentary R4.5.6 written by Reviewer 4. This commentary was not properly satisfied. Number of ions needed to charge the membrane-capacitor up to the voltage about 70mV is 5 orders of magnitude less that the total number of ions in the neuron. So, any realistic, i.e., approximate values of ionic concentrations cannot determine the voltage. As a consequence, the authors need this artificial number of impermeable anions, 97%.

AA3.3. The capacitor-charge relation C*U = Q occurs in the cable equation (in differential form), and is used by basically all neuron models that we are aware of. We are convinced that this is an applicable way of modeling membrane potential dynamics: Charge passing through the membrane in the form of ionic currents leads to a charging up of the membrane. Also, it is well known that the number of ions that need to cross the membrane in order to charge it up by, say, an action-potential worth of millivolts is indeed several orders of magnitudes smaller than the number of ions in the intracellular space, and thus does not cause any noteworthy change in any of the ionic concentrations on a short time scale (with possible exceptions for Ca2+, since the intracellular baseline concentration is orders of magnitudes lower than for Na+, K+ and Cl-).

The fact that we need a large number of impermeable anions is not related to the use of the charge-capacitor equation, but to the fact that our model does not explicitly include absolutely all ion species present in the intracellular space. There is basically no doubt that the saline solutions in the intra- and extracellular space always remains extremely close to electroneutral, since any deviance from electroneutrality will be prevented by very strong electric forces (see e.g. book by Grodzinsky 2011: Grodzinsky, Alan J. *Fields, forces, and flows in biological systems*. Garland Science, 2011).

Using table values for the most commonly modeled ion species, Cl-, Na+, K+ and Ca2+ (e.g., from table 2.1 in Somjen 2001 (10 mM Na+, 125 mM K+, 6.6 mM Cl-, and a very small Ca2+ concentration), the only way that the intracellular space can be electroneutral, is if there is additional negative charge there. This charge can be realized in the forms of negatively charged macromolecules (proteins, actin filaments, possibly with large negative valencies) or other, smaller ions (HCO3-, OH-) not included in the model. Some of the latter could possibly permeate the membrane in real life, but we did not include their dynamics in the model, so they were "static" there.

Furthermore, we do not think that a large fraction (like 97%) of anions that are not chloride is unrealistic. On the contrary, values that we find in textbooks suggest that there is indeed a large concentration of anions inside neurons. In table 7-1 in the book Principle of Neural Sciences (Kandel, Schwartz, Jessell), the concentration of organic anions in the squid giant axon is enormous:

Table 7-1 Distribution of the Major Ions Across a Neuronal Membrane at Rest: the Giant Axon of the Squid

Species of ion	Concentration in cytoplasm (mM)	Concentration in extracellular fluid (mM)	Equilibrium potential[1] (mV)
K^+	400	20-75	
Na^+	50	440+55	
Cl^-	52	560-60	
A^- (organic anions)	385	——	

[1] The membrane potential at which there is no net flux of the ion species across the cell membrane.

Furthermore, if we do a google picture search on "concentration + organic anions + neuron + table" a number of tables pop up, and many of them give us suggestions that are in the same ballpark (we found 100 mM or 154 mM). In a textbook exercise that popped up in this search (see below) an anion concentration (154 mM) is used to illustrate (among other things) the principle of electroneutrality in the intracellular bulk solution, i.e., the principle that we computed the anion concentration from in our model. Hence, we do not think that there is anything unreasonable with the modeling assumptions that we have made regarding the capacitor-charge relation or the intracellular concentration of static anions.

Table 1-1 gives the ion concentrations for neuron A. Refer to this table to answer the question below.

Ion	[ ] Inside (mM)	[ ] Outside (mM)
Na^+	10	160
K^+	150	11
Cl^-	6	159
$Anion^-$	154	12

The intracellular and extracellular solutions of neuron A result in:

- ○ Osmotic balance between the intracellular and extracellular solutions
- ○ Electrical neutrality in the intracellular and extracellular compartments
- ○ Electrical neutrality in only one cellular compartment
- ○ Neuron A being at rest
- ○ None of the above

RR3.4/RR3.12. The limitation of the model has been described in the text. The model has not been changed.

AA3.4/AA3.12. In both these cases, the claimed "limitation" was that the model does not account for lateral diffusion of ions, or diffusion into an external bath solution. Therefore, it is not suited to model slice experiments (RR3.4), or letting K+ diffuse out of the system and in that way (possibly) relieving the neuron from its K+ dependent state of depolarization block (RR3.12). It is true that we did not change the model accordingly, but the "limitations" that were referred to are not really limitations but rather a modeling choice. It is fairly straightforward to run the model with various kinds of open boundary conditions, and we tested this in a few simulations that we did not include in the manuscript. Any modeling choice will call for a new analysis and calibration of the model. To keep the "story short", we did not want to present several different versions of the model. In the current implementation, our objective was not to model slice experiments or diffusion into a bath solution. Our objective was to model DC potentials in tissue, and the modeling choices were made accordingly.

RR3.6. Still, the simulations can hardly remind any experiments.
RR3.8. So experiments are mentioned only in the Introduction. The problem of the work is that it is not targeted to any certain experiments.

AA3.6/AA3.8. We think that we have been rather clear about the limitations in this regard. The model is a fairly abstract one, with the pros and cons that comes with an abstract model. We do not know how to tailor it to represent a specific experimental context in any detail. We believe that the model is still useful in the sense that it can (1) shed light on and give qualitative insight into a phenomenon that cannot be addressed with other models, and (2) can inspire and be used as a tool in future modeling studies.

RR3.10. So, the model describes "the average activity (all cells) in a unit-piece of tissue". Are neurons distinguished by types, like excitatory and inhibitory, at least? If even not, how one can use the proposed set of equations for modeling synaptically interacting populations of neurons? This is the main goal, isn't it? With the present equations one can simulate only spikes from a single neuron that represents a population of neurons. However, there is a big gap between the spikes and a firing rate that governs the synaptic activity.

AA3.10. Our goal was never to model synaptically interacting populations of neurons, and we have never claimed so. This kind of (domain-type) model would probably not be a good choice if this was the goal. Our goal was instead to answer the question: For neurons in tissue firing in a specific way, which different mechanisms will contribute to the generation of DC potentials. Neuronal transmembrane sources, glial transmembrane sources, or extracellular diffusion?

In the re-revised version, we have slightly expanded this analysis, by decomposing the transmembrane sources/sinks into the different contributors (various ion channels, stimulus currents, or ion pumps). We have added two new figures on this as supplementary material.

RR3.19. Still, it seems that the constructed model has only parts of functionality that is necessary to simulate phenomena involving concentration gradients and synaptic interactions of different type neurons (see my complains **RR3.2 and RR3.10)**, so it is not quite self-consistent.

AA3.19. By self-consistent, we did not mean that it contains absolutely all relevant functionality, but that it (unlike many other models) has an internally consistent relationship between the variables that it does contain. You are right that the model is rather minimal – it does not account for neuronal diversity and synaptic interactions between neurons, it is very limited in a spatial and morphological sense, and it contains only a small subset of the membrane mechanisms present in real tissue. But then again, every model is a simplification, and again the question boils down to whether the model can be used to gain insight into important features of DC potentials, and mechanisms causing them. And again, we believe that it does.

Reviewer #4:

This is probably one of the best revisions that I have seen in my career as a reviewer. I particularly want to thank the authors for their extensive and well thought answers on all my questions, even those that are no longer relevant for the present version of the manuscript.

One of the main criticisms of the previous version of this paper was that it was to much a model for the model paper, which will restrict the audience to only a relatively small group of actual modelers. The authors have now made a revision, which still presents the same interesting model, but they have focused much more on real neuroscience questions then on speculations of for what the model could be used for. The main issue is now on (small) slow potentials in nerve tissue (and a bit on depolarization block).

As any good paper raises more questions than it can answer, a have a few points to make. Except for one question out of interest, they do not demand additional experimental work, and most can probably be easily handled at a textual level.

-- In the description and the potential use of the model (abstract and introduction/discussion lines 459-477) it might be helpful for potential users to explicitly state that the model is restricted to polarized neurons organized in a layered palisade way: typically, hippocampus regions. Five-layered cortex is not impossible but would be quite a programming challenge!

We have added the following sentence to the Discussion (lines 492-494):

We note that the spatial heterogeneity of the model restricts it to brain regions where neurons are organized in layers with all neurons having the same spatial orientation (such as, e.g., hippocampus).

-- At several points in the paper the authors generate and discuss depolarization block. I fully agree with those points, but it should be mentioned that the model is not optimized to handle that topic. In my experimental experience it is rather hard to induce depolarization block in hippocampal pyramidal neurons. The model does not

contain somatic calcium channels coupled to strong potassium channels that produce slow and fast after hyperpolarization and spike frequency adaptation. They limit firing rate and prevent depolarization block to a large extend. It is not a main point of the paper but should be discussed.

We have added the following discussion in the Results section (lines 248-267):

We note that there are two main ways in which a neuron can be driven into depolarization block. The perhaps most well-studied scenario is that when the neuron fires so fast that it does not have time to repolarize properly between two consecutive action potentials. The action potential amplitude will then gradually decrease and tend to zero, repolarization will eventually fail, and the membrane potential will approach some depolarized equilibrium value. Neurons often have mechanisms (such as Ca2+ channels coupled to afterhyperpolarizing K+ channels) that limit firing rates and prevent them from entering firing-rate dependent depolarization block. An alternative scenario is when the depolarization block is caused by an increase in the extracellular K+ concentration to values high above baseline due to, e.g., intense firing or impairment of the Na+/K+ pump [Binder 2009].
An enhanced extracellular K+ concentration will (i) depolarize the neuron due to an increased K+ reversal potential, and at the same time, (ii) make repolarizing K+ currents weaker due to the reduced K+ gradient. Jointly, these two effects can put the neuron in a state where voltage-activated ion channels remain open, and the neuron gradually dissipates its concentration gradients approaching a depolarized equilibrium where it is unable to fire. The first type of depolarization block can be explored using models that do not model ion concentrations explicitly (see, e.g., bianchi2012, dovzhenok2012), while the second type can only be studied in models that include ion concentration dynamics [Oyehaug2012,Sætra2020] and is the one that we observe here.

-- The authors induce activity in the neuron either by current injection or by synaptic activation. Current injection is done by transferring K+ (or Na+/Cl-) ions from the extra- to the intra-cellular compartment. This guarantees conservation of ions but invalidates electroneutrality in both compartments. Is that physically realistic and acceptable? Please comment.

The input used in our simulations, i.e., the transfer of ions across the membrane, is equivalent to what happens when an ion channel opens: Ions move across the membrane to charge or discharge it. As such, the input current is equivalent with the other ionic membrane currents.

The model was constrained to preserve bulk-electroneutrality, not strict electroneutrality. Deviance from electroneutrality occurs at the membrane, and is consistent with the membrane potential through the capacitor equation: "*phi_m = Q/C_m*" (cf. eq. 18 in the manuscript), where the charge *Q* in a given compartment is interpreted as the membrane charge (since the bulk should be electroneutral), *phi_m* is the membrane potential of that compartment, and *C_m* the membrane capacitance. This assumption is implicit in most existing neural models (cable based, Hodgkin-Huxley, LIF neurons etc). Our model is different only in the sense that it explicitly keeps track of the ion concentrations that constitute this charge.

In our model, the membrane charge, *Q*, is found by summing up the charge associated with the various ionic concentrations within the compartment. The rationale is: If the compartment contains a nonzero charge, this charge sits exclusively on the membrane (and is thus reflected in *phi_m*), while the bulk is electroneutral.

We note that the same rationale is used also in the extracellular compartments, but the situation is somewhat more complicated there, since the compartment charge in the extracellular space must be consistent with the summed (outside) membrane charge of both the neuron and glial cell. This was ensured by the modeling framework, and follows from the current-anti-symmetry criterion (eq. 24).

We have added the following line in the Methods section (lines 792-793) to clarify this:

In this aspect, the stimulus was equivalent to a current through an open ion channel.

Stimulation is always followed by re-equilibration of the ions and we noticed in our own (unpublished) models that due to the small Cl- currents this can be a very slow process.

I appreciate the way synaptic input is implemented, as it avoids the problem mentioned above. There is, however, also here one peculiarity and that relates to the Mg2+ dependence of the NMDA synapse. Mg2+ is not present in the model so the authors set it to a fixed level of 1 mM. As it is membrane impermeable, it will undergo huge changes in concentration with the volume changes of the EC compartment. In reality [Mg] is often high enough to saturate the binding; it does not seem to be implemented that way here. It could also be that in the current studies (except the depolarization block) the voltage never reaches values sufficient to release the block. Using a glutamate synaps would have avoided this inconsistency and lead to the same conclusions.

To avoid this inconsistency, we have replaced the NMDA synapse with an AMPA synapse. Consequently, Figure 7 is updated and we have made changes to the “Synaptic current” section in Methods (page 34). As you expected, the new synapse leads to the same conclusions in the Results section as before.

-- In lines 407-417 the authors compare the depolarization block state with a Donan equilibrium. To the best of my knowledge the latter one comes about because of the non-permeable (an)ions inside the cell, after concentration gradients have faded. But the resulting membrane potentials can never produce extracellular gradients. That must be brought about by the ion pumps that are still working. It would be a simple and interesting run to stop the pumps and observe the resulting Donan potentials in all compartments.

We agree and have removed "Donnan" from the text. We now refer to this state as (lines 434-435):

an equilibrium determined by the intrinsic dynamics of the system after the neuron had entered depolarization block

We followed your suggestion, and made a simple run where we stopped the pumps and co-transporters. We did not include this simulation in the manuscript, but attach the results here. As the figure to the right shows, it then took the neuron about 5 min to reach the resulting Donnan-equilibrium.

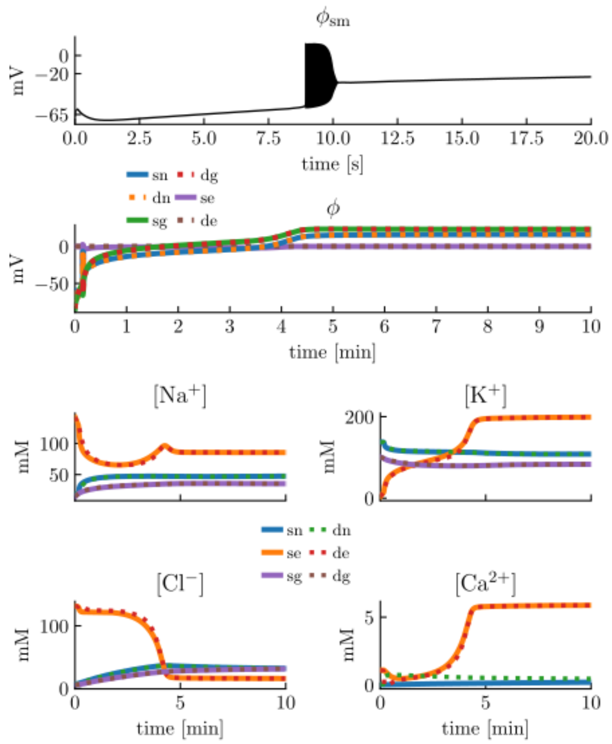

-- The (preliminary)results on the slow potentials and their contributors are also quite interesting and promising. Could the authors also indicate what the relative contribution of the different pumping components is?

We have now decomposed the transmembrane sources/sinks into the different contributors (various ion channels, stimulus currents, or ion pumps) and added this as supplementary material (S1 Fig and S2 Fig). The Figures are referred to on lines 369-373, 376, and 395.
